# TRPM8 channel inhibitor-encapsulated hydrogel as a tunable surface for bone tissue engineering

**DOI:** 10.1038/s41598-021-81041-w

**Published:** 2021-02-12

**Authors:** Tusar Kanta Acharya, Satish Kumar, Nikhil Tiwari, Arijit Ghosh, Ankit Tiwari, Subhashis Pal, Rakesh Kumar Majhi, Ashutosh Kumar, Rashmita Das, Abhishek Singh, Pradip K. Maji, Naibedya Chattopadhyay, Luna Goswami, Chandan Goswami

**Affiliations:** 1 School of Biological Sciences, National Institute of Science Education and Research (NISER)-Bhubaneswar, Jatni, Khurda, 752050 Odisha India; 2 Homi Bhabha National Institute, Training School Complex, Anushakti Nagar, Mumbai, 400094 India; 3 School of Biotechnology, Kalinga Institute of Industrial Technology, Patia, Bhubaneswar, 751024 India; 4 School of Chemical Technology, Kalinga Institute of Industrial Technology, Patia, Bhubaneswar, 751024 India; 5 Division of Endocrinology and Center for Research in Anabolic Skeletal Target in Health and Illness (ASTHI), Central Drug Research Institute (CDRI), Council of Scientific and Industrial Research (CSIR), Lucknow, 226031 India; 6 Department of Polymer and Process Engineering, Indian Institute of Technology Roorkee, Saharanpur Campus, Paper Mill Road, Saharanpur, 247001 Uttar Pradesh India; 7 AcSIR, CSIR-Central Drug Research Institute Campus, Lucknow, 226031 India

**Keywords:** Cell biology, Biomaterials

## Abstract

A major limitation in the bio-medical sector is the availability of materials suitable for bone tissue engineering using stem cells and methodology converting the stochastic biological events towards definitive as well as efficient bio-mineralization. We show that osteoblasts and Bone Marrow-derived Mesenchymal Stem Cell Pools (BM-MSCP) express TRPM8, a Ca^2+^-ion channel critical for bone-mineralization. TRPM8 inhibition triggers up-regulation of key osteogenesis factors; and increases mineralization by osteoblasts. We utilized CMT:HEMA, a carbohydrate polymer-based hydrogel that has nanofiber-like structure suitable for optimum delivery of TRPM8-specific activators or inhibitors. This hydrogel is ideal for proper adhesion, growth, and differentiation of osteoblast cell lines, primary osteoblasts, and BM-MSCP. CMT:HEMA coated with AMTB (TRPM8 inhibitor) induces differentiation of BM-MSCP into osteoblasts and subsequent mineralization in a dose-dependent manner. Prolonged and optimum inhibition of TRPM8 by AMTB released from the gels results in upregulation of osteogenic markers. We propose that AMTB-coated CMT:HEMA can be used as a tunable surface for bone tissue engineering. These findings may have broad implications in different bio-medical sectors.

## Introduction

There is a surge in bone-related disorders primarily linked with old-age, unhealthy life-style and bio-medical conditions like bone cancer, osteoporosis, etc. which cause decay and/or loss of bones^1^. Many of these bone-related problems would benefit from the availability of immediate therapeutic intervention with near-functional bone, either as an autograft or allograft. However, such replacement possibilities are limited due to insufficient supply of appropriate materials coupled with potential biomedical risks involving undesired immune response, infection, non-compatibility, genetic mismatch and tissue rejection^2,3^. The alternative strategies holding promise in this context include bone tissue engineering using differentiated and/or stem cells as therapeutic strategies^3^. In this case, major problems mentioned above can be avoided, but a serious bottleneck is efficient adhesion, growth, differentiation of stem cells and finally mineralization by these cells^4^. This is mainly because stem cells require specific yet compatible surfaces for their adhesion, growth and differentiation. Similarly, bio-mineralization is a complex process by which soluble Ca^2+^ is converted to insoluble Ca^2+^-complex matrix through cellular metabolism^5^. Ideally the stem cell therapy for each patient must be tailor-made. Processes and biomaterials supporting adhesion, growth and differentiation of stem cells to osteoblasts and promote bone mineralization, can overcome challenges present in bone tissue engineering.

Bone formation and maintenance are primarily regulated by the osteogenic activity of osteoblasts and remodeling activity of bone-resorbing osteoclasts^6^. Pre-osteoblasts and Mesenchymal Stem Cells (MSC) differentiate into mature osteoblasts and undergo mineralization which provides the rigidity of the bone^7^. Notably, all these biological events leading to bone mineralization are stochastic in nature, time-consuming and difficult to influence in a reproducible manner, both in in vitro and in vivo conditions^8^. To overcome this problem, it is desirable to utilize artificial surface that serves two primary purposes: first, the surface must be compatible for growth and differentiation of stem cells. The surface must allow methodologies that are suitable to control the stochastic biological events to a more defined and directed process leading to desired growth of osteoblasts followed by mineralization. In addition, the surface should be low-cost and easily available.

Hydrogels contain hydrophilic polymeric chains that form three-dimensional structure. Therefore, hydrogels can mimic the extracellular matrix which allows cells to make proper contacts and functionally active. Hydrogels can also be structured to become tunable which in turn can influence temperature, pH, elasticity and other biochemical properties. Due to these advantages, cell behavior such as adhesion, migration, proliferation, and differentiation can be controlled in real-time by maintaining the appropriate combination of stimuli as well as hydrogel composition and architecture^9^. Despite these promises, so far effective materials that can differentiate stem cells to osteoblasts and enhance bio-mineralization in a tunable manner has not been developed.

It is well established that Ca^2+^-signaling plays a vital role in the process of osteogenesis, yet the key players involved in this entire process is not well known^10^. Never-the-less, ion channels provide molecular routes for Ca^2+^-influx as well as initiating points targeting signaling pathways and gene expression that are stochastic otherwise, yet critical for osteoblast functions^11^. However, it needs identification of important ion channels as cellular targets for osteogenesis and precise activation or inhibition of these channels, preferably in spatiotemporal manner and in a dose-dependent manner.

Recently several members of TRP channels (a group of non-selective cationic channels) have been reported to be expressed endogenously in osteoblasts and involved in bone homeostasis^11^. Thermosensitive TRP channels, namely TRPV1, TRPA1, and TRPM8 have been reported to be functionally present in human and rat odontoblasts and their inhibition leads to blockage of temperature-induced calcium entry into odontoblasts^12^. TRPV1 and TRPV4 are endogenously expressed as functional ion channels in both osteoblasts and osteoclasts. The pharmacological blockage of TRPV1 prevents differentiation of osteoclast and osteoblast^13^. Mutations in TRPV4 are linked to different skeletal deformities^14^. Similarly, the TRPM8 transcript has been detected in human osteoblast-like cells, namely in MG-63, SaOS2, U2OS and murine pre-osteoblast cell line MC3T3-E1^15^. However, protein level expression and functional role of TRPM8 have not been demonstrated in osteoblasts. In this context, recent reports have identified higher level expression of TRPM8 mRNA and protein in osteosarcoma rather than normal bone tissue^16^. Recently we have reported that TRPM8 is a vertebrate-specific ion channel suggesting that it is probably involved in bone homeostasis^17^. All these reports suggest that TRPM8 might have endogenous expression in osteogenic cell lines and thus TRPM8 can be targeted for potential bone-tissue engineering.

In this work, we explored the expression and function of TRPM8 in different osteoblasts as well as in Mesenchymal Stem Cells. Our data suggest that long-term pharmacological modulation of TRPM8 is sufficient for triggering osteogenesis in vitro, and specifically TRPM8 inhibition enhances osteogenesis and mineralization via up-regulation of osteogenic genes. For that purpose, we used CMT:HEMA hydrogel as a surface. Previously we have reported that this hydrogel provides optimum surface for cells with osteogenic as well as with non-osteogenic properties^18,19^. This hydrogel is also biologically neutral as it does not induce any unwanted side effects. Here, we used CMT:HEMA as a scaffold for sustained release of drugs modulating TRPM8 ion channel activity. The combination of CMT:HEMA surface and long-term pharmacological modulation of TRPM8 provides a platform for effective bone-tissue engineering using Mesenchymal Stem Cells.

## Results

Bone development and maintenance depend on Ca^2+^-signaling and intracellular Ca^2+^-loading. Ca^2+^ channels present in osteoblasts are key regulators of bone Ca^2+^-homeostasis, and function of few such channels is known. The presence and role of cold-sensitive TRPM8 channel has not been explored in osteoblasts till date, therefore we explored the endogenous expression and function of TRPM8 in osteogenic cells and evaluated the effect of TRPM8 modulation on osteoblasts followed by application of TRPM8 modulation for in vitro bio-mineralization.

### TRPM8 ion channel is endogenously expressed in osteoblasts

**Figure 1:**
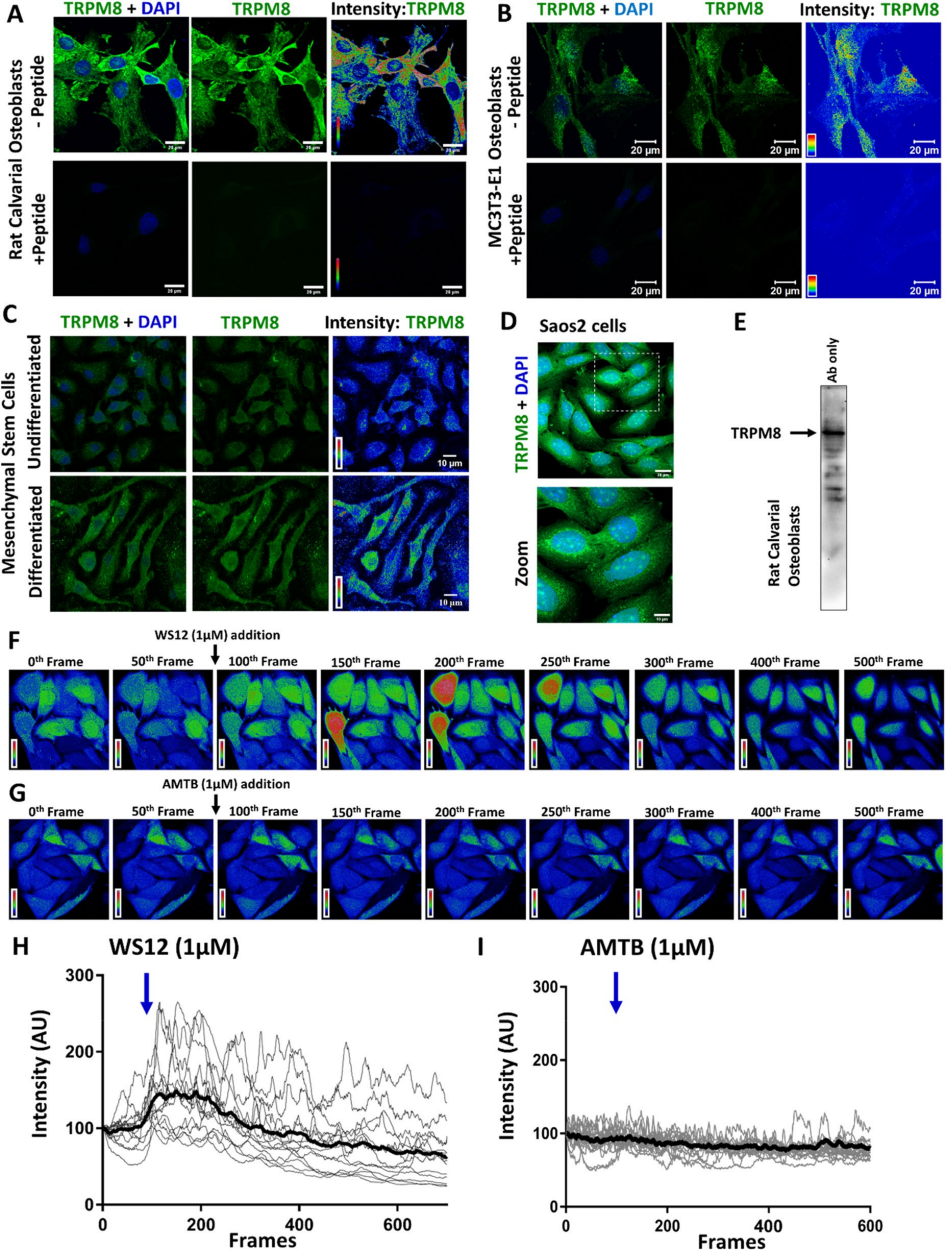
Functional TRPM8 is endogenously expressed in osteoblasts. **(A)** Confocal images of Rat Calvarial Osteoblasts (RCOs) cultured for 7 days in differentiation medium and probed for endogenous TRPM8 (green) using anti-TRPM8 antibody in absence (upper panel) or in presence of its antigenic peptide (lower panel). (**B)** Confocal images of mice pre-osteoblasts MC3T3-E1 cultured in differentiation media for 7 days and probed for endogenous TRPM8 (green) using anti-TRPM8 antibody in absence (upper panel) or in presence of its antigenic peptide (lower panel). (**C)** Confocal images of bone marrow derived Mesenchymal Stem Cells (MSCs) undifferentiated (upper panel) and differentiated to osteoblasts (lower panel) are shown. Higher TRPM8 signal is observed in differentiated MSCs. (**D)** Confocal images of human osteosarcoma cell line Saos2 depicting TRPM8 expression (green) are shown. (**E)** Western blot of RCOs cultured for 7 days in differentiation medium are shown. Blot was probed for endogenous TRPM8 using anti-TRPM8 antibody. (**F,G)** Time series images of Ca^2+^-intensity in live Saos2 cells. Cells were loaded with Fluo-4-AM and fluorescence intensity is represented in pseudo color (red and blue indicating highest and lowest intensity respectively). Calcium imaging was performed at the speed of 2 Frames per second. At 100th frame, cells were treated with TRPM8 activator WS12 (1 µM) **(F)** or inhibitor AMTB Hydrate (1 µM) **(G)**. (**H,I)** Quantification of Fluo-4-AM intensity (in AU) over 500 frames, with the initial value normalized at 100%. Increased fluorescence intensity is observed in most cells treated with WS12, which declines gradually over time. TRPM8 inhibitor (AMTB) doesnot alter Fluo-4-AM intensity over time **(G)**.

Rat calvarial osteoblasts (RCOs, primary pre-osteoblasts) were cultured for 7 days in differentiation medium and probed for endogenous TRPM8 levels. Confocal imaging revealed the presence of TRPM8 throughout the RCOs (Fig. 1A, upper panel). TRPM8 signal was completely blocked upon pre-incubating the TRPM8 antibody with its antigenic peptide, indicating specificity of the antibody used (Fig. 1A, lower panel). Western blotting of RCOs revealed a TRPM8 specific band at the expected molecular weight of ~ 129 kDa (Fig. 1E). The endogenous expression of TRPM8 was also probed in mice pre-osteoblasts MC3T3-E1. High-level expression of TRPM8 throughout the cells is observed upon culturing them in differentiation media for 7 days (Fig. 1B). Specificity of TRPM8 staining was further confirmed by the absence of signal in cells stained with TRPM8 antibody pre-incubated with its antigenic peptide (Fig. 1B, lower panel).

The major source of osteoblasts in bones is the Mesenchymal Stem Cells (MSCs) residing in bone marrows. Therefore, we probed for the expression of TRPM8 in undifferentiated MSCs and differentiated osteoblasts obtained from mice. Endogenous TRPM8 was present in both MSCs and MSC-derived osteoblasts, however intensity profiling of TRPM8 showed higher levels of TRPM8 in differentiated osteoblasts (Fig. 1C). High-level TRPM8 expression was also detected in human osteosarcoma cell line Saos2 (Fig. 1D).

### TRPM8 channel modulates intracellular Ca^2+^-dynamics in osteoblasts

In order to probe for the functionality of TRPM8 in bone cells, Ca^2+^-imaging was performed using Fluo4-AM loaded into Saos2 cells (Human osteosarcoma cell line). TRPM8 activator WS12 (1 µM) added at the 100^th^ frame, was able to induce sharp spike in intracellular Ca^2+^-levels in most of the osteoblasts. Few osteoblasts remained insensitive to WS12 (Fig. 1F,H). This indicated that TRPM8 is functional in Saos2 cells. However, addition of TRPM8 inhibitor (AMTB 1 µM) did not alter the intracellular calcium levels in Saos2 cells as reflected by insignificant changes in Fluo4 intensity with respect to time (Fig. 1G,I).

### TRPM8 is a key molecule regulating differentiation and maturation of osteoblasts

**Figure 2:**
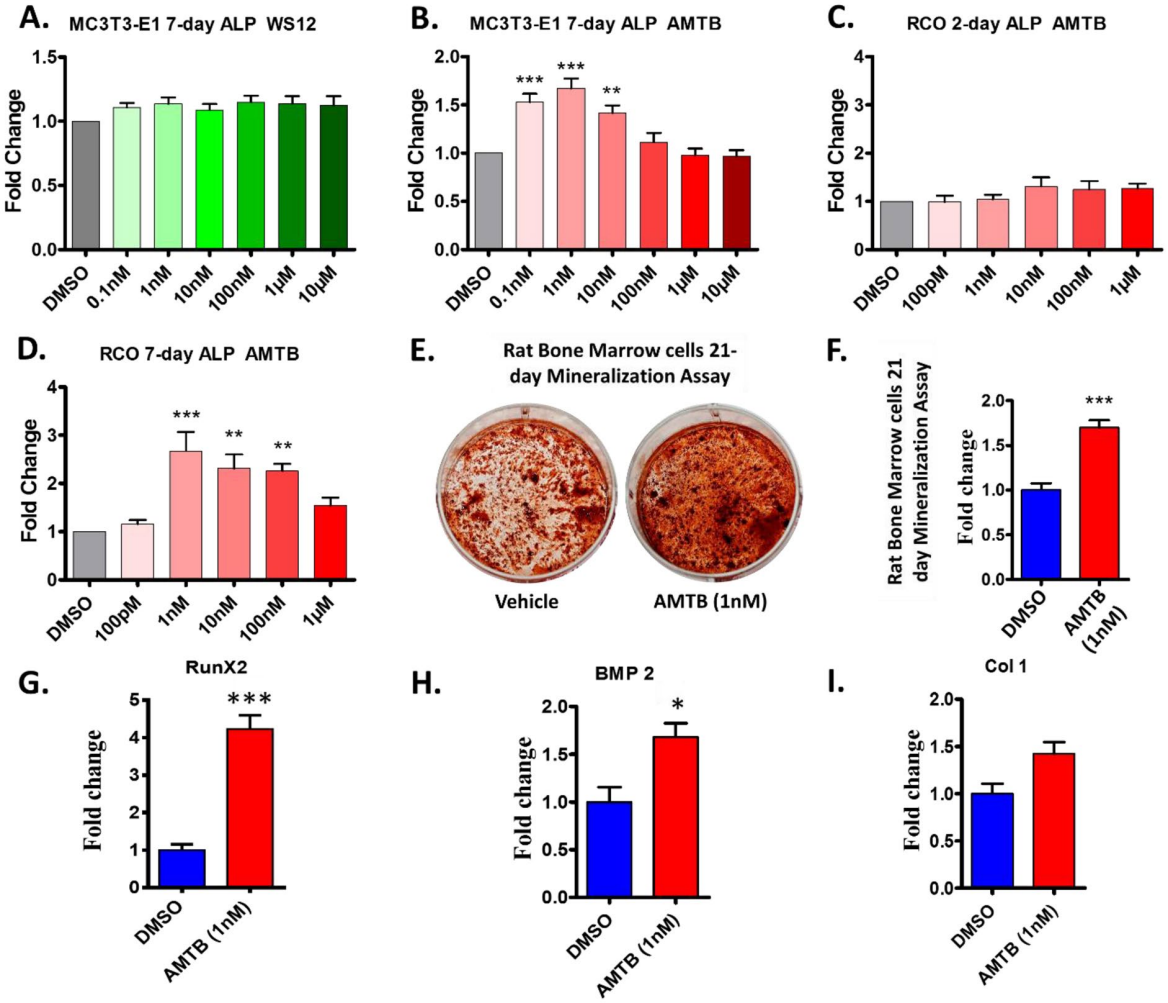
TRPM8 inhibition enhances osteoblast differentiation and mineralization. **(A,B)** Bar graphs represent 7-day Alkaline Phosphatase (ALP) activity of mice pre-osteoblasts MC3T3-E1 treated with increasing concentrations of TRPM8 activator WS12 (**A**, n = 6) or inhibitor AMTB Hydrate (**B**, n = 6). (**C,D)**. Bar graphs represent ALP activity of Rat Calvarial Osteoblasts (RCOs) grown for 2 days (**C**, n = 6) and 7 days (**D**, n = 4) in presence of TRPM8 inhibitor AMTB Hydrate in increasing concentrations. (**E)** Photographs represents mineralization nodules of Rat Bone Marrow cells grown for 21 days in presence of DMSO control or AMTB Hydrate (1 nM) as detected by Alizarin Red staining. (**F)** Corresponding quantification of Alizarin Red intensity is shown as a measure of bone mineralization due to AMTB Hydrate (1 nM) for 21 days (n = 3). (**G,I**) Graphs depict the RT-PCR fold change values of osteogenic genes upon TRPM8 inhibition by AMTB Hydrate for 48 h. Transcript levels (n = 3) of RunX2 increases by ~ 4.2 fold, Bmp2 increases by ~ 1.8 fold and Col1 increases by ~ 1.5 fold. ANOVA test, p values: *   < 0.05, **   < 0.01, ***   < 0.001.

Next, we explored the role of TRPM8 channel in the differentiation of murine pre-osteoblast cell line MC3T3-E1. Cells were cultured in the presence of TRPM8 activator WS12 or TRPM8 inhibitor AMTB in increasing concentrations (0.1 nM to 10 μM). DMSO-treated cells were taken as control (vehicle) group. Cells were fixed by 4% Paraformaldehyde (PFA) after 7 days of culture. Extent of differentiation was checked by Alkaline Phosphatase (ALP) assay as ALP activity increases upon differentiation of pre-osteoblasts into mature osteoblasts. Activation of TRPM8 by WS12 (all concentrations tested) does not have any effect on differentiation of MC3T3-E1 cells (Fig. 2A). AMTB at very low concentrations (0.1 nM, 1 nM and 10 nM) significantly increases differentiation of pre-osteoblasts into mature osteoblasts (Fig. 2B).

Since TRPM8 inhibition increased differentiation and maturation of MC3T3-E1 cells, we probed for the effect of TRPM8 inhibitor on the differentiation status of primary rat calvarial osteoblasts (RCOs) ex vivo. RCOs are the source of pre-osteoblasts in neonatal rats, which later differentiate into osteoblasts that constitutes the skull of matures rats. Primary rat calvarial osteoblasts (RCOs) were cultured in the presence of TRPM8 inhibitor AMTB in increasing concentrations (100 pM to 1 μM). DMSO-treated cells were treated as the control (vehicle) group. Cells were fixed by 4% PFA after 2 days or 7 days of culture. Extent of differentiation was checked by ALP assay. TRPM8 inhibitor AMTB (1 pm to 1 μM) did not show much difference in ALP activity after 2 days of culture (Fig. 2C). However, consistent with the differentiation pattern obtained with MC3T3-E1, significant increment in differentiation of RCOs was observed after 7 days of culture in presence of AMTB (at 1 nM, 10 nM and 100 nM concentrations) (Fig. 2D). In fact, AMTB (1 nM) is sufficient to increase the differentiation of RCOs by ~ 2.5 fold (Fig. 2D). These results confirm TRPM8 as a key signaling channel involved in differentiation of osteoblasts.

### TRPM8 inhibition enhances mineralization of rat bone marrow osteoblasts

Since the differentiation assays suggested that TRPM8 inhibition helps in differentiation of osteoblasts, we performed a 21-day mineralization assay using Rat Bone Marrow cells (RBMs) to mimic in-vivo like conditions. RBMs were cultured in mineralization media in presence or absence of AMTB (1 nM) for 21 days. Cells were fixed with PFA and stained with Alizarin Red dye to analyze the extent of mineralization. Qualitative microphotographs of the mineralization plates (Fig. 2E) and subsequent quantification by CPC extraction method indicated that TRPM8 inhibitor AMTB (1 nM) increases mineralization by ~ 1.5 fold (Fig. 2F).

### TRPM8 inhibition induces differentiation of rat calvarial osteoblasts by increasing the expression of osteogenic genes

To elucidate the mechanism of osteogenesis induction triggered by TRPM8 inhibition, we cultured RCOs in differentiation media in presence or absence of AMTB (1 nM) and quantitatively determined mRNA levels of osteogenic genes: runt-related transcription factor 2 (RunX2), bone morphogenic protein-2 (Bmp2) and collagen type-1 (Col1) following an optimized protocol described before^20^. Glyceraldehyde-3-phosphate dehydrogenase (GAPDH) was used as the internal control in this study. AMTB (1 nM) treatment increases mRNA level of RunX2 in differentiating RCOs by ~ 4.2 fold, Bmp2 by ~ 1.8 fold and Col1 by ~ 1.5 fold after 48 h of AMTB treatment (Fig. 2G–I). All these data suggest that TRPM8 inhibition by pharmacological means can be adapted for enhanced bio-mineralization.

### CMT:HEMA hydrogel surface properties

We have previously described the synthesis and characterization of a polysaccharide based hydrogel CMT:HEMA as an effective biocompatible surface which can act as a suitable material for bone tissue engineering^18^. Recently we have also shown that this hydrogel is biocompatible for skin cells as well as murine derived Mesenchymal stem cells^19^. This hydrogel doesnot have any adverse effects when applied on the skin of live rats, indicating its suitability for in vivo applications^19^. Therefore, we have characterized this hydrogel as a suitable surface and medium for sustained release of drugs. We also used this hydrogel for pharmacological targeting of TRPM8 ion channel in order to achieve bio-mineralization.

**Figure 3:**
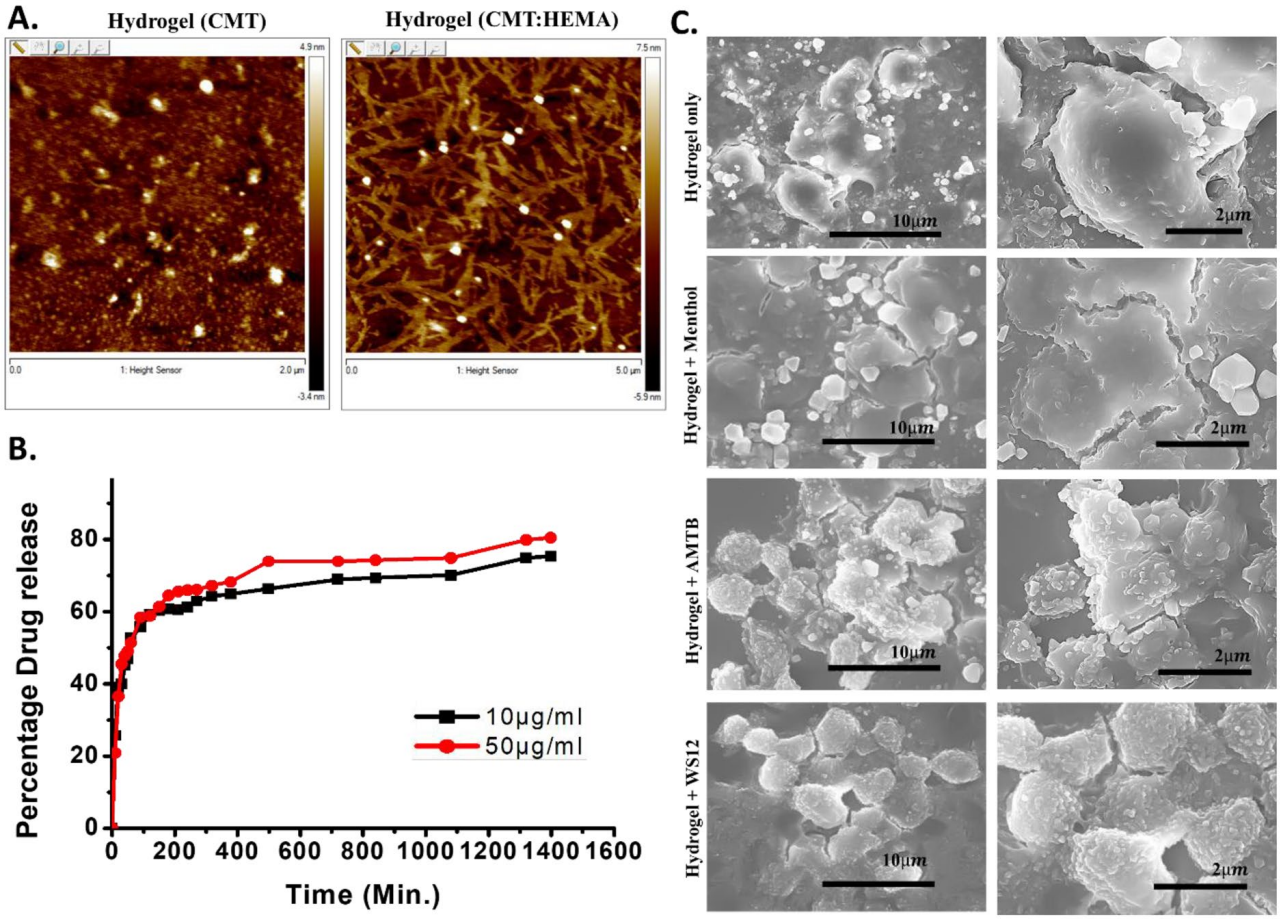
Hydrogel (CMT:HEMA) as a suitable surface for drug coating and growing of Mesenchymal Stem Cells. **(a)** Shown are the Atomic Force Microscopic images of CMT only and CMT:HEMA. CMT:HEMA provides a surface with optimum network and stiffness suitable for growth of Mesenchymal Stem Cells. C-0 corresponds to pure carboxymethyl tamarind while C-10 corresponds to Hydroxyethylmethacrylate incorporated carboxymethyl tamarind hydrogel (i.e. CMT-HEMA hydrogel). (**b)** Release pattern of AMTB drug from hydrogel (CMT:HEMA) are shown. (**c)** Scanning Electron Microscopic images of Mesenchymal Stem Cells grown on hydrogel (CMT:HEMA) only and coated with different drugs modulating TRPM8 ion channel are shown in left side. Magnified images of same or different view field are shown in right side.

CMT:HEMA has uneven rough surface in dry conditions and with bigger pores in wet conditions. Such morphology is ideal for growth and adhesion of cells with osteogenic properties. In order to get further details of the surface morphology, we tested only CMT as well as CMT:HEMA by Atomic Force Microscopy. The AFM data reveals that CMT has ovoid morphology of the nanostructure polymer (Fig. 3A). HEMA grafted CMT shows the fibrous morphology of the matrix. The change in the AFM images of CMT before and after modification indicates the structural changes in the matrix. Better matrix coherency achieved after HEMA modification is evident from AFM micrographs. Both CMT have granular morphology, and this is drastically converted into coherent and near co-continuous morphology in HEMA modified CMT. This is in line with the observation from FTIR analysis.

### CMT:HEMA hydrogel is suitable for encapsulation and release of ion channel specific drugs

We encapsulated the CMT:HEMA hydrogel with TRPM8 activator WS12 at varying concentrations (1 nM, 10 nM, 100 nM, 1 µM) and TRPM8 inhibitor AMTB (1 nM, 10 nM, 100 nM, 1 µM). The WS12 absorbance spectra coincide with the spectra of its solvent DMSO, hence couldnot be distinguished via UV spectrophotometry. However, release of AMTB was observed over time with around 60% release by 5 h (300 min) and up to 80% release even after 1 day (1600 min) (Fig. 3B).

An ideal hydrogel intended for therapeutic drug delivery application should not interact either with model drug or solvent. Here we have analyzed the effect of different concentration of AMTB on encapsulation and loading efficiency (%) of hydrogel (Supplementary Fig. 1a). The percentage encapsulation and loading efficiency increases with increase in concentration. At higher concentration (100 µM) it was 93.79 and 4.02% respectively. Similar trends in case of ciprofloxacin loaded on ultrasonic hydrogel was reported recently^21^. These observations may be attributed to the porous nature of hydrogel (as observed in AFM analysis).

### CMT:HEMA hydrogel act as suitable surface for growth of mesenchymal stem cells

We explored whether Mesenchymal Stem Cells can grow on CMT:HEMA as well as drug-coated CMT:HEMA gels. We observed that Mesenchymal Stem Cells grow well on these surfaces, yet with different morphologies and roughness in the surface (Fig. 3c). The MTT assay indicates that none of these drug-coated hydrogels are toxic and cells grow well on these surfaces. In fact, cell survival is better when grown on the drug-coated gels rather than on the glass surface with the same concentration of drugs (Supplementary Fig. 1b-c).

### CMT:HEMA encapsulated with TRPM8-specific compounds regulate cellular morphology

**Figure 4:**
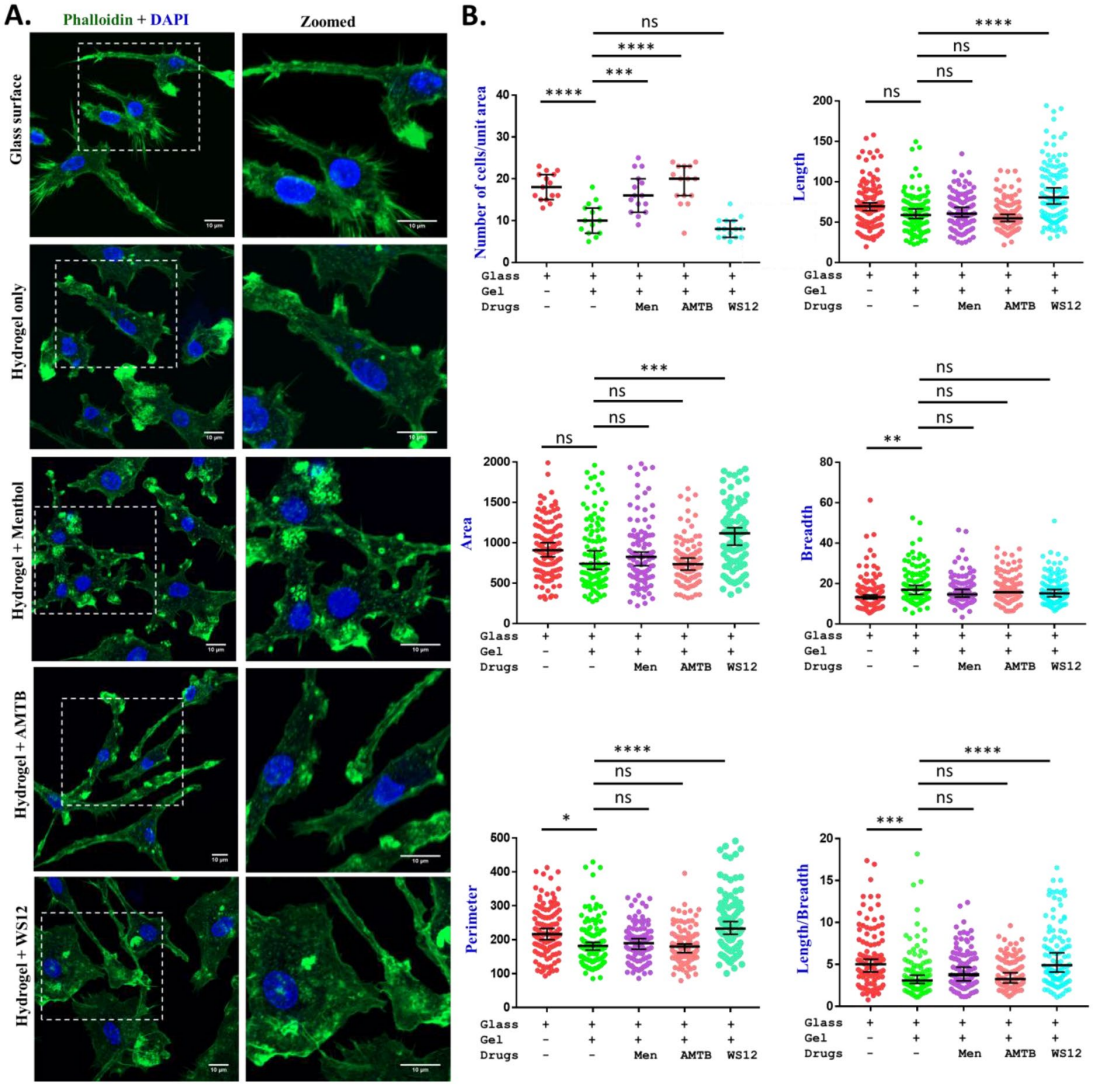
Hydrogel-mediated drug release alters the morphology of bone marrow derived mesenchymal stem cell population. BM-MSCPs were isolated and grown on only glass, only hydrogel or on hydrogel-coated with different drugs affecting TRPM8 ion channel. **(A)** Morphology of cells grown on different surface are shown. Cells were stained with Phalloidin (green) and DAPI (blue). **(B)** Different morphology parameters of the cells grown on different surface are shown. The statistical values are: ANOVA test, p values: *   < 0.05, **   < 0.01, ***   < 0.001, ****  < 0.0001.

Differentiation of MSCs to osteoblasts and subsequent mineralization involves specific changes in the morphology. Such morphological changes are pre-requisite for actual mineralization. We explored if CMT:HEMA hydrogel is able to induce morphological changes in BM-MSCPs. Therefore, BM-MSCPs were grown on the glass surface, hydrogel coated coverslips as well as in hydrogels coated with menthol, WS12, and AMTB. Cells were stained with Phalloidin-488 as well as with DAPI and images were acquired (Fig. 4A). We quantified number of cells grown per unit area. We also quantified the morphological parameters such as length, breadth, area, and parameter are quantified from 100 cells on each coverslips (Fig. 4B). While number of cells grown on unit area on CMT:HEMA hydrogel alone is less than only glass, the number of cells grown is significantly higher when either Menthol (TRPM8 activator) or AMTB (TRPM8 inhibitor) is used (Fig. 4B, upper left panel). This suggests that CMT:HEMA alone as well as drug-coated CMT:HEMA can be used for desired number of cells on unit area (discussed later). There is no significant difference in the cell length, width, cell perimeter or even cell area in most cases (Fig. 4B). However, presence of WS12, an activator of TRPM8 in the CMT:HEMA causes increased length, increased perimeter, increased cell area but no change in cell width. In contrast, presence of AMTB, an inhibitor of TRPM8 in the CMT:HEMA causes no change in cell length, width, perimeter, and area when compared to the CMT:HEMA only (Fig. 4B). This data suggests that CMT:HEMA with or without TRPM8-specific drugs affect cell adhesion and cell migration differently (discussed later).

### CMT:HEMA encapsulated with TRPM8-specific compounds induce differential ROS signaling

**Figure 5:**
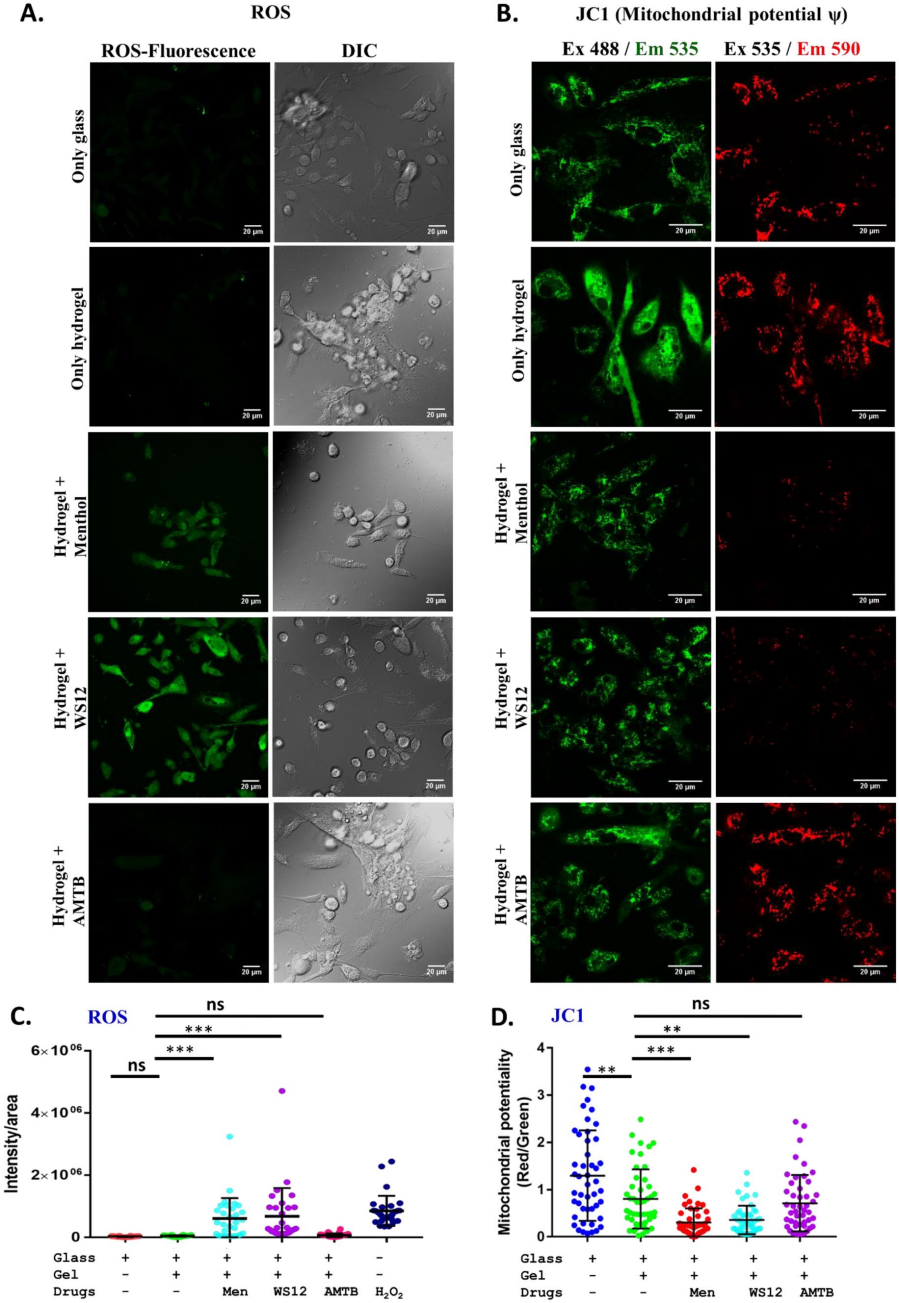
Hydrogel coated with TRPM8 activators but not with inhibitor specifically stimulates ROS signaling by decreasing mitochondrial oxidative potential. **(A,B)** Fluorescence microscopic images from cells grown on only glass, only hydrogel or the same hydrogel coated with different activators and inhibitor are shown. ROS signal (green in left panel) and DIC images of the cells are shown in left side **(A)**. Similarly, mitochondrial oxidative potential analyzed by JC1 dye is shown in right side **(B)**. JC1 fluorescence emission at 590 nm (red panel, indicative of high oxidative potential) is reduced in cells when grown on hydrogel coated with TRPM8 activators. (**C,D)** Quantified values representing the fluorescence values from individual cells grown on different surface as shown in figure **(A,B)**. The statistical values are as follows: ANOVA test, p values: **  < 0.01, ***  < 0.001, *ns* non-significant.

Reactive Oxygen Species (ROS) signaling is important for bone mineralization^22^. Therefore we analyzed the extent of ROS produced by Mesenchymal stem cells when grown on CMT:HEMA only or CMT:HEMA coated with TRPM8 specific drugs. We used H_2_O_2_ (20 μM) as a positive control, as it is known to generate free radicals and generate ROS. We quantified the relative amount of ROS produced in each condition using Image-J. We did not observe any difference in fluorescence intensity of ROS in cells grown on glass and only hydrogel (Fig. 5A,C). This confirms that the cells that are grown on Hydrogel are producing a negligible amount of ROS. On the other hand, hydrogels coated with menthol and WS12 are producing modest amount of ROS that is tolerable by cells (Fig. 5A,C). In contrast, AMTB-coated hydrogel produces basal level of ROS. This data also demonstrate the CMT:HEMA can be fine-tuned for ROS production applicable to other cells also.

### CMT:HEMA encapsulated with TRPM8-specific compounds affect mitochondrial membrane potential

We explored if TRPM8 activation can cause changes in the mitochondrial membrane potential. For this purpose, Mesenchymal Stem Cells were grown on hydrogel alone as well as hydrogel-coated with TRPM8 specific activator or inhibitor for 24 h. Subsequently cells were labelled with ratiometric dye JC-1. Subsequently image intensity (Red and Green) was quantified by Image J. The Red intensity (Ex535/Em590) represents higher membrane potential (Fig. 5B,D). In the presence of CMT:HEMA hydrogel Mitochondrial potential is significantly decreased as compared with only glass as surface. However, in the presence of TRPM8 activator Menthol (10 μM) or WS12 (1 μM), mitochondrial potential was significantly decreased as compared to CMT:HEMA alone. TRPM8 inhibitor, AMTB (1 μM) did not alter the mitochondrial potential in BM-MSCPs and retains its membrane potential similar to the CMT:HEMA only (Fig. 5B,D).

### CMT:HEMA encapsulated with TRPM8-specific inhibitor enhances bone differentiation and mineralization

**Figure 6:**
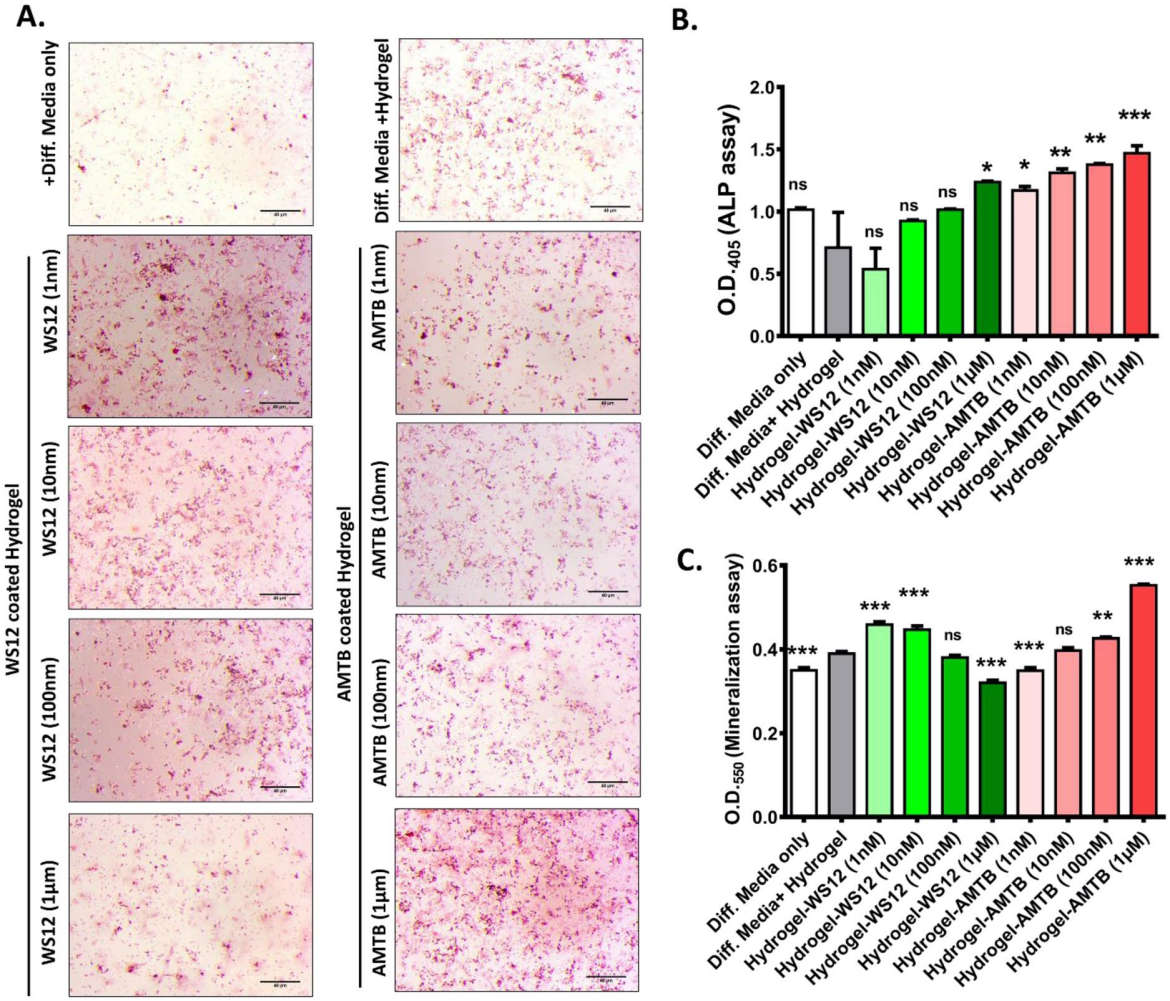
Hydrogel (CMT:HEMA)-mediated release of TRPM8 agonist and antagonist modulates osteoblasts functions. (**A)** Photographs (upper panel) represents mineralization nodules of Mesenchymal Stem Cells (MSCs) grown for 15 days in absence or presence of hydrogel (CMT:HEMA) as detected by Alizarin Red staining (n = 3). The lower panel photographs represent the extent of mineralization nodules formed when cells were grown on WS12 or AMTB encapsulated CMT:HEMA hydrogel. Scale bar 40 µm. (**B,C)** Bone marrow-derived Mesenchymal Stem Cell population (BM-MSCPs) grown on these hydrogels or hydrogels-encapsulated with AMTB or WS12 reveals dose-dependent differentiation **(B)** and mineralization **(C)** (n = 3). The significance values are as follows: ANOVA test, p values: *   < 0.05, **   < 0.01, ***   < 0.001.

BM-MSCPs grown on plates coated with hydrogels containing TRPM8 specific drugs were viable and proliferated well (data not shown). ALP assay performed after 7 days of culture show a dose-dependent increase in ALP activity in cells grown on AMTB-coated CMT:HEMA hydrogel when compared to cells grown on only CMT:HEMA hydrogel in presence of differentiating factors (BGP, LAA and Dexamethasone) (Fig. 6B). However, WS12 coated hydrogels did not significantly affect ALP activity. None of these hydrogels decreased ALP activity, showing that they are suitable for targeting BM-MSCPs as well as Osteoblasts. This was in full agreement with the results obtained upon application of TRPM8 activator or inhibitor alone, as described above. The hydrogel alone did not increase or decrease ALP activity, suggesting that the results obtained above are the effects of slow and local release of TRPM8 modulators only (Fig. 6B).

Further, we performed mineralization assay after 15 days of culturing BM-MSCPs on hydrogel coated plates in the presence of differentiating factors (BGP, LAA, and Dexamethasone). We observed that very low concentrations of WS12 coated hydrogels (1 nM and 10 nM) induce increase in mineralization, while higher concentrations either donot affect mineralization (WS12; 100 nM) or decrease mineralization significantly (WS12; 1 µM) (Fig. 6C). Opposite effect was observed in case of AMTB coated Hydrogels. Very low concentration of AMTB in hydrogel either decrease mineralization (AMTB 1 nM) or donot affect mineralization (10 nM), while higher concentration of AMTB coated hydrogel increase mineralization significantly (100 nM and 1 µM) (Fig. 6C). Higher number of Alizarin-red stained mineralized nodules are visible in WS12 (1 nM) coated CMT:HEMA hydrogel and in AMTB (1 µM) coated CMT:HEMA hydrogel but not in only CMT:HEMA hydrogel (Fig. 6A) confirming the results obtained after extraction of Alizarin Red by CPC extraction (Fig. 6C). The expression of osteogenic markers such as Osteopontin and Osteocalcin show significant changes when grown with these TRPM8-modulatory drugs (Supplementary Fig. 2, 3).

Taken together, we demonstrate that endogenous and functional TRPM8 is present in osteoblasts and TRPM8 modulators can be utilized pharmacologically for enhanced osteoblast differentiation and mineralization by CMT:HEMA hydrogel-based surfaces.

## Discussion

Bone structure and integrity are fine-tuned by continuous large-scale anabolism and catabolism events depending on the relative number as well as activity of osteoblasts and osteoclasts respectively^23^. Mineralization by osteoblasts is a complex and slow process, stochastic in nature and dependent on many intracellular and extracellular factors. However, selective ion channels can overcome these bottlenecks and TRPM8 channel turns out to be a key regulatory molecule involved in the differentiation of BM-MSCPs to osteoblasts and subsequent bio-mineralization process. These findings allowed us to use BM-MSCPs directly for the enhanced biomineralization in vitro, yet in a tunable manner using CMT:HEMA hydrogel and long-term pharmacological inhibition of TRPM8 in a slow-release and dose-dependent manner.

We have previously shown CMT:HEMA as a suitable biomaterial for successful adhesion and growth of osteoclasts and keratinocytes^18,19^. We now demonstrate that the same hydrogel is suitable for osteoblasts and Mesenchymal Stem Cells. The CMT:HEMA hydrogel has an undulating surface, porous structure and fibrous connectivity, properties that are suitable for drug encapsulation and cell growth. Osteoblasts prefer surfaces that are relatively hard for proper adhesion^24^. The results suggest that in case of WS12-encapsulated hydrogel, the number of cells in unit area is low, yet the attached cells have increased length, increased perimeter and increased area. Together the data suggest for a better “cell spreading” event on the WS12 containing hydrogel surface. This is justified by the fact that cell adhesion and cell spreading depends on the effective focal adhesion points, which is very much dependent on the Ca^2+^-dynamics and Ca^2+^-signaling events. This could also explain the presence of lesser number of cells per unit area on CMT:HEMA surface. However the total mineralization levels on CMT:HEMA is similar to that on a glass surface, suggesting that this hydrogel itself (without any drug encapsulation) supports basal mineralization rates. Taking advantage of the fact that CMT:HEMA represents nano-fibrous structure and is also suitable for encapsulation of pharmacological drugs and their sustained release, we achieved enhanced bio-mineralization in CMT:HEMA encapsulated with TRPM8 inhibitor AMTB. The extent of mineralization is tuneable, mediated by the long-term release of TRPM8-specific pharmacological compounds in a dose-dependent manner. In this context, it is important to consider that CMT:HEMA alone supports basal level of osteoblast growth and mineralization. However, CMT:HEMA mediated sustained release of TRPM8-specific drugs enhance differentiation, mineralization of osteoblasts. This is also by one-time application of these drugs in the hydrogel which bypasses the requirement of administrating the drugs in large quantities’ in every alternate day for bone healing.

In this work, we demonstrate that cells with osteogenic properties respond to agonist for TRPM8 and results in rise in intracellular Ca^2+^. In contrast, inhibition of TRPM8 results in no change in Ca^2+^-levels, at least quickly. In case of TRPM8 inhibition, the intracellular Ca^2+^-levels reduce gradually and in a slow manner. Such rise and fall of intracellular Ca^2+^ levels matches well with the general Ca^2+^ oscillation pattern observed in osteogenic cells which is relatively slower than other excitatory cells such as neurons^25^. The Ca^2+^-imaging experiment conduced here measures total change in intracellular Ca^2+^-levels in single cells and from such changes‚ firing pattern of single ion channels can be predicted but cannot be concluded. In addition to endoplasmic reticulum, other intracellular organelles such as mitochondria and lysosome also play a major role in the regulation of intracellular Ca^2+^-homeostasis and Ca^2+^-metabolism which are essential for bone mineralization. Mitochondria act as a major Ca^2+^-storage and transport organelle during osteoblast development^5^. Mitochondria act as localized [Ca^2+^] buffering units and modulate intracellular Ca^2+^ levels by initiating feedback-inhibition or activation mechanisms relevant for bone cell functions^26^. Bone cells consume a lot of energy and the production of ATP is largely dependent on calcium. The mitochondrial F_1_F_0_-ATP synthase is stimulated by Ca^2+^^27^, uptake of which changes the mitochondrial membrane polarity. In the same manner, it was recently shown that Reactive Oxygen Species (ROS) plays a major role in both osteoclasts and osteoblasts^28^. Cells produce more ROS when it comes under stress and ROS-signaling plays a significant role in cell differentiation process, while excessive ROS production may cause disruption in homeostasis^29^. In these contexts, CMT:HEMA encapsulated with different TRPM8 modulators offers materials where both mitochondrial, lysosomal functions as well as ROS levels which can be finely controlled.

TRPM8 is not the only Ca^2+^-permeable channel which is expressed in bone cells and importance of other ion channels in osteogenesis and bio-mineralization cannot be ruled out. However, as a proof-of-principle we have explored the relative expression of TRPV4 (another ion channel that is known to express in several cells with osteogenic properties) when cells are grown on different surfaces with or without TRPM8-specific pharmacological agents. We found that there is significant upregulation of TRPV4 expression when cells are grown on only hydrogel surface as compared to glass surface (data not shown). On contrary, there is no change in the TRPV4 expression when cells were grown on hydrogel with TRPM8-specfic drugs” as compared to glass as a surface. Therefore, it suggests that expression of other channels may or may not be affected by prolonged activation or inhibition of TRPM8 and such understandings need further detailed work.

In this work, we demonstrate that TRPM8 ion channel offers a suitable molecular route for bio-mineralization. TRPM8 has added advantages over any other ion channels for several reasons. First, TRPM8 is not essential as knockout animals also survive without major bone problems. This may also suggest that pharmacological inhibition of TRPM8 will not cause any major or deleterious physiological problems in vivo. Besides, the drug release by the hydrogels will produce a more localized effect rather than systemic effect. At physiological temperatures (i.e. 35–37 °C), there are high possibilities of spontaneous activation of other thermosensitive ion channels such as TRPV3 or TRPV4 (activation temperature ~ 27 °C and ~ 37 °C). This essentially means that in the standard culture conditions/physiological conditions, TRPM8 will not be activated unless influenced by pharmacological compounds. Therefore, TRPM8 offers a more specific molecular route that can be utilized for osteogenesis by pharmacological means and without having any deleterious side effects. The pharmacological agents used in this process are easily available, often from natural sources and are of relatively low cost.

We observed certain differences in the mineralization extent when cells were activated by drugs applied to the media directly or cells were exposed to the drugs through hydrogel-mediated slow and local release. Approximately 1.5-fold mineralization is achieved in 21 days by total bone marrow cells (from rats) when AMTB is directly added to 1 ml cell culture media (making the final concentration at 1 nM). AMTB needs to be freshly added in every 2 days (during media change), summing up to 10 nM total (Total 10 time addition during the entire experimental period). On the other hand, 1 µM AMTB encapsulated hydrogel (10 µl) present in 1 ml media causes similar level of mineralization in 15 days (using BM-MSCPs from murine bone marrow). Therefore one-time application of AMTB-encapsulated CMT:HEMA hydrogel enhances the efficacy of drugs by almost 100 times and reduces the time required by several days, making it time and cost-effective. The stage of the cells also seems to play a role in case of TRPM8-induced mineralization process. For example, RCO at day 2 remain non-responsive to AMTB, but the same RCO show dose-dependent response at day 7.

In recent times, bone-related problems are common, and this scenario enforces the generation of artificial bone at low cost. TRPM8 inhibitor, namely AMTB at very low concentrations could provide an opportunity for their therapeutic use in enhancing osteogenesis and could be relevant for treatment of different bone-related disorders such as osteoporosis where more bio-mineralization is required. The CMT:HEMA, being a slowly degrading hydrogel can be a useful tool in slow but sustained delivery of TRPM8 inhibitors at sites of injury. Such prospects need further investigation. Taken together, all these suggest that inhibition of TRPM8 affects bone formation and such findings may have broad implications in bio-medical implications.

## Materials and methods

### Materials

CMT:HEMA hydrogel was synthesized as described before^18^. Cell culture-related reagents such as α-MEM, 1 × PBS, Trypsin–EDTA, FBS, Penicillin–Streptomycin and Amphotericin B were purchased from HIMEDIA (India). McCoy’s 5A media was purchased from PAN BIOTECH (Germany). Rabbit polyclonal antibody raised against extracellular loop region of TRPM8 and a specific blocking peptide (SDVDGTTYDFAHC) were was purchased from ALOMONE Labs (Israel). Anti-rabbit–HRP antibody was purchased from GE, USA. Alexa flour 488-labelled anti-rabbit antibody, DAPI, Alexa 488-labelled Phalloidin, H_2_DCFDA, JC1 dye, MitoTracker Green and LysoTracker Red were purchased from MOLECULAR PROBES (USA). The TRPM8 modulatory drugs WS12, AMTB and Menthol were purchased from Sigma (USA). Hydrogen Peroxide was purchased from MERCK (USA).

### AFM

Atomic force microscope (Bruker Dimension Icon AFM, USA) operated in scan-assist mode for morphological and surface feature analysis. The images were then analyzed by NanoScope analysis software, version 1.4.

### Coating of CMT:HEMA hydrogel with TRPM8 modulators and evaluation of drug release

In vitro drug release efficacy of hydrogel was examined by methods described before with some modifications^30^. Briefly, hydrogel film was immersed into 5 ml of AMTB solutions in a glass beaker for 24 h for equilibrium encapsulation of AMTB inside the hydrogel matrix. After 24 h hydrogel was taken out from drug solution and the extra drug was removed by careful drenching. Percentage Drug dissolution study of the drug with respect to time was examined by Type-II apparatus (Electrolab dissolution tester, EDT-08Lx), at temperature 37 °C at 50 rpm in drug dissolution buffer. At predetermined time interval 2 ml of drug solution was taken out from dissolution basket and replaced by fresh dissolution buffer. The drug concentration in withdrawn solution was determined by taking the absorbance at 274 nm (λ_max_ of AMTB) in UV–Vis spectrophotometer.

### Animal procurement and primary cell isolation

Animals used for this work were procured either from NISER or CDRI animal house facility. This study was approved by Committee for the Purpose of Control and Supervision of Experiments on Animals (CPCSEA) and Institutional Animal Ethics Committee. All methods were performed in accordance with the relevant guidelines and regulations of Committee for the Purpose of Control and Supervision of Experiments on Animals (CPCSEA) and Institutional Animal Ethics Committee (IAEC) of NISER and CDRI (CDRI/IAEC/2013/34, NISER/SBS/IAEC/AH-55 & NISER/SBS/IAEC/AH-77) . Adult Sprague–Dawley (SD) rats (4–8 weeks old) were sacrificed by cervical dislocation. Bone marrow was flushed out from tibia with culture media (α-MEM supplemented with 10% FBS) as mentioned before^20,29,30^. After passing through 70 μm cell strainer (SPL Labs), bone marrow cells were collected and seeded (CORNING Corporation). SD pups (0–2 days old) were sacrificed by decapitation. Calvaria was carefully taken out, washed with PBS, muscles, and blood were removed. The calvarial fragments were then subjected to enzymatic digestion by using 10 mg/ml collagenase and dispase solution (SIGMA ALDRICH) in 37 °C water bath for 1.5 h with gentle shaking. Cells were collected after passing the digested solution through a 70 μm cell strainer. Cells were cultured in α-MEM supplemented with 10% FBS till confluence.

Adult BALB/c mice (4–6 weeks old) were sacrificed by CO_2_ asphyxiation followed by cervical dislocation. BM-MSCPs were isolated as per procedures with minor modifications. Bone marrow was flushed out from tibia and femur with chilled culture media (α-MEM supplemented with 10% FBS). After passing through 70 μm cell strainer (SPL Labs), bone marrow cells were subjected to RBC lysis and centrifuged. Cells were resuspended in α-MEM supplemented with 10% FBS and plated in 90 mm dishes (CORNING Corporation).

After 1 day of incubation, the non-adherent cells (containing primarily hematopoietic cells) are removed, the adherent cells are washed twice with PBS and cultured up to a week in CO_2_ incubator in α-MEM supplemented with 10% FBS. These cells are enriched in BM-MSCPs, and proliferate rapidly. After 1–2 passages, sufficient cells were visually examined to be fibroblast-like morphology and are obtained for experimentation. These cells, primarily represent BM-MSCPs and are subsequently seeded on hydrogel coated or uncoated coverslips or plates for functional studies.

### Cell culture

MC3T3-E1 cells (subclone 14, ATCC, USA) were maintained in α-MEM media (SIGMA ALDRICH) supplemented with 10% Fetal Bovine Serum and Penicillin Streptomycin (HIMEDIA). Saos2 cells (ATCC, USA) were maintained in McCoy`s media supplemented with 10% FBS and penicillin streptomycin. Mineralization media was prepared by adding 10 mM β-glycerophosphate, 100 μg/ml L-ascorbic acid and 10 nM Dexamethasone (SIGMA) to the α-MEM culture media^20,31,32^.

### Growing cells on hydrogel-coated surface

The prepared drug-gel combination was applied to 18 mm coverslip, coating it evenly and left for air drying. Simultaneously the coverslips were UV-irradiated in 12 well culture plates. Upon drying, mouse bone marrow-derived Mesenchymal Stem cells were seeded in each well (approx. 1 × 10^5^ cells/well) over drug-coated coverslips and left to adhere for at least 24 h. After sufficient number of cells were adhered to the coverslips, media supplemented with L-Ascorbic Acid and beta-glycerophosphate was added, discarding the old media completely. Fresh media was added in every 4 days keeping half amount of the old media in well. For ALP the experiment was continued for 7 days and experiment was continued for 15 days for mineralization.

### Alkaline phosphatase assay

ALP assay media was prepared by adding 100 μg/ml L-ascorbic acid and 10 mM β-glycerophosphate to culture media^20,31,32^. Equal number of cells were seeded in 96-well plates (CORNING) and cultured for 3 or 7 days in the presence or absence of drugs. Subsequently, media was aspirated and cells were washed with PBS. Cells were lysed by subjecting the plate to repeated freeze–thaw cycle and ALP substrate (SIGMA ALDRICH) was put in equal quantity in each plate. After 3 h of incubation, spectrophotometric reading was taken at 405 nm in an ELISA plate reader (VarioSkan, photometry mode). CMT:HEMA hydrogel was used as a carrier for release of TRPM8-specific modulators. These gels were coated on 24-well plates and murine BM-MSCPs were seeded with or without differentiation media. After 7 days, ALP assay was performed as per procedure described above.

### Immunocytochemistry

Cells were cultured on 12 mm coverslips in 24 well plates. After differentiation, cells were fixed with 2% Paraformaldehyde (PFA, Sigma Aldrich) for 15 min at room temperature (~ 25 °C). PFA was then aspirated from the wells and cells were washed thrice with PBS, permeabilized with 0.1% Triton X-100 in PBS for 5 min and blocked with 5% Bovine Serum Albumin (BSA) for 30 min. Cells were then probed with the anti-TRPM8 antibody (Alomone Labs, Israel) overnight and detected by Alexa Fluor-488 conjugated secondary antibody (Invitrogen). DAPI (2.5 μg/ml) was used as a counterstain to visualize the nucleus. For confirming the specificity of the antibody, TRPM8 epitope-specific peptide (Alomone Labs) was used. The primary antibodies were pre-incubated with this blocking peptide. PFA-fixed cells were stained with Phalloidin 488 (Invitrogen) for 2 h and cells are visualized by using confocal microscopy (Zeiss LSM 800).

### Quantification of reactive oxygen species (ROS)

Quantification of ROS generation was performed by microscopic analysis using intracellular oxidation of a cell-permeable dye, i.e. 2′, 7′-dichlorodihydrofluorescein diacetate (H_2_DCFDA) (Invitrogen). The H_2_DCFDA measures reactive oxygen species (ROS) activity within the cell. Approximately 10,000 bone marrow-derived mesenchymal stem cell population was seeded on glass cover slips and on hydrogel-coated cover slips. Cells were incubated with 10 μm H_2_DCFDA at 37 °C for 20 min. In a similar manner 50 μm H_2_O_2_ (MERCK) treated cells were taken as a positive control. Subsequently the fluorescence signals of ROS were observed under confocal microscope (FV 3000). Images were processed in ImageJ software.

### JC-1 staining

Mitochondrial potential in Mesenchymal stem cells were analyzed by labelling the cells with 5,5,6,6-tetrachloro-1,1,3,3-tetraethylbenzimidazolylcarbocyanine iodide (JC-1) cationic dye. The JC-1 dye exhibits potential-dependent accumulation inside the mitochondria and show potential-dependent shift in fluorescence emission properties (from green range 525 nm to red 590 nm region). After growing the cells in TRPM8-specific drug-containing hydrogel, cells were incubated with JC-1 (5 μM) for 30 min at 37 °C. Cells on coverslips were placed in to the live cell chamber and images were acquired by confocal microscope (FV 3000) in the respective excitation and emission regions as described before^33^.

### Live cell Ca^2+^-imaging

For live-cell imaging of basal Ca^2+^-levels, Fluo-4 AM-based method was used as described before^34^. Saos2 cells were plated in 24 mm coverslips in 35 mm tissue culture dishes (CORNING) and cultured in differentiation media for 3 days. Cells were treated with or without pharmacological activators for 2 days and then taken out of incubator after 45 min treatment with 0.02% PluronicF-127 (Thermo Fisher Scientific) and 5 μM Fluo-4 AM (Molecular Probe). Live cell imaging was then performed in a Zeiss LSM 780 confocal microscope.

### qRT-PCR analysis

Quantitative real-time polymerase chain reaction (qPCR) was performed to determine the relative expressions of osteoblast and osteoclast specific genes in drug-treated RCOs. GAPDH was used as internal control. The design oligonucleotide primers were based on published cDNA sequences using the Universal Probe Library (Roche Applied Sciences)^20^. Primer sequences against specific genes are listed in (Table 1). All cDNA were synthesized with Revert Aid cDNA synthesis kit (Fermentas, Austin, USA) using 2 μg total RNA. SYBR green chemistry was employed to perform quantitative detection of relative expression of mRNA levels of these genes using a Light Cycler 480 (Roche Molecular Biochemicals, Indianapolis, USA) as described previously^20^.

**Table 1:** Primer sequences of various genes used for qRT-PCR.

Gene name	Primer sequence	Gene accession number
RunX2	F-5′ CCACAGAGCTATTAAAGTGACAGTG 3'	NM_053470
	R-5′ AACAAACTAGGTTTAGAGTCATCAAGC 3'	
BMP2	F-5′ CGGACTGCGGTCTCCTAA 3'	NM_007553.2
	R-5′ GGGGAAGCAGCAACACTAGA 3'	
Col1	F-5′ CATGTTCAGCTTTGTGGACCT 3'	NM_053304
	R-5′ GCAGCTGACTTCAGGGATGT 3'	
GAPDH	F-5′ TTTGATGTTAGTGGGGTCTCG 3'	NM_017008
	R-5′ AGCTTGTCATCAACGGGAAG 3'	

### SDS-PAGE and western blot analysis

RCOs were harvested after 7 days of culture in differentiation medium and gel samples were prepared by boiling the samples for 5 min with Laemmli buffer supplemented with protease inhibitor cocktail (SIGMA ALDRICH). These samples were analyzed by 10% SDS-PAGE followed by Western blot analysis as described before^35^.

### Scanning electron microscopy

Morphological analysis of cells grown on hydrogel film was analyzed by scanning electronic microscopy (SEM CARL ZEISS neon 40). Briefly, BM-MSCPs were derived and cultivated from bone marrow as described above. BM-MSCPs were cultivated on hydrogel film for 48 h, subsequently, cells were washed three times with phosphate buffer saline (1X PBS, pH-7.2) and fixed by PBS containing 2.5% glutaraldehyde (SIGMA ALDRICH USA) and 3% PFA. Hydrogel film containing cells were lyophilized (CHRIST alpha 1–2 LD Plus) for 24 h and sputter-coated with gold before SEM analysis.

### Mineralization assay

Mineralization assay was performed by seeding the equal number of cells in 12-well plates and cultured in mineralization media for 21 days in presence or absence of any TRPM8 modulatory drugs. After 21 days, media was aspirated, cells were washed with PBS and equal amount of Alizarin Red-S (ARS) dye was added to each well. After 30 min of incubation, the ARS dye was washed with water and micrographs were taken to qualitatively assess the extent of mineralization. The extent of mineralization was quantified by extracting ARS dye by Cetyl Peridynium Chloride (CPC, MP Biomedicals) and spectrophotometric reading was taken at 595 nm as mentioned above. CMT:HEMA hydrogel releasing TRPM8 modulators were coated on 24-well plates and murine bone marrow-derived Mesenchymal Stem Cells (BM-MSCP) were seeded with or without differentiation media. After 15 days, mineralization assay was performed as per procedure described above.

### Encapsulation efficiency and loading efficiency of hydrogel

Encapsulation and drug loading efficiency of AMTB in CMT:HEMA hydrogel were analyzed by reported method with minor modification^36^. Briefly, AMTB solution of varying concentration were prepared and equal amount of CMT:HEMA hydrogel were immersed in those AMTB solutions. After 24 h of AMTB-loaded hydrogel were taken out carefully. Absorbance of supernatant (free AMTB) was analyzed by using UV–vis spectrophotometer (AGILENT CARRY-100). The Free AMTB concentration in supernatant was calculated from the standard curve of AMTB (10-100 µM). The encapsulation efficiency (%) and drug-loading efficiency was determined by equations described before^37^.

### Cell proliferation analysis by MTT assay

Proliferation of mouse bone marrow derived mesenchymal stem cells in presence of different concentration of TRPM8 activator or inhibitor and these drugs-encapsulated in hydrogels were investigated by MTT assay. Briefly 8000, mesenchymal stem cells are seeded in 96 well plate and incubated for 48 h. Subsequently, cells were treated with different concentration of AMTB and WS-12 in solution, or activator/ inhibitor-coated hydrogel (1 nm to 1 µM). Cells were maintained at 37 °C in CO_2_ incubator, cells grown without drug and hydrogel was taken as a control. After that, supernatant was carefully discarded and 200 µl of MTT solution (0.1 mg/ml) was added in each well and further incubated for 3 h at 37 °C for crystal development. After formation of formazan crystals, MTT dissolving dye was added in each well for dissolving crystal, and absorbance were recorded by using ELISA plate reader at 570 nm.

### Staining of osteopontin and osteocalcin

Mouse bone marrow derived Mesenchymal stem cells were cultured over the hydrogel coated with different concentration of WS12 and AMTB in the presence of mineralization media. After 14 days of culture, the cells were fixed with 2% PFA. The cells were stained with antibodies specific for Osteopontin (Novus Biologicals, NB110-89062) and osteocalcin (Novus Biologicals, MAB1419-SP). Alexa-Fluor-594 was used for the secondary antibody staining.

### Statistical tests

Data is represented as graphs with Mean ± SEM. One-way ANOVA test with Dunnett’s post hoc test was done. The significance of each test has been reported by the means of p-value.

## Supplementary Information

Supplementary Figures.
