# Supplementary material for: TRPM8 channel inhibitor-encapsulated hydrogel as a tunable surface for bone tissue engineering
Source: Sci Rep. 2021 Feb 12;11:3730. doi: 10.1038/s41598-021-81041-w (PMC7881029; doi:10.1038/s41598-021-81041-w)
Supplement: Supplementary file 1 — Supplementary Figures. [file 41598_2021_81041_MOESM1_ESM.docx]

**Supplementary information:**

**TRPM8 channel inhibitor-encapsulated hydrogel as a tunable surface for bone tissue engineering**

**Tusar Kanta Acharya^1,2,♣^, Satish Kumar^3,4,♣^, Nikhil Tiwari^1,2,^*****, Arijit Ghosh^1,2,^*****, Ankit Tiwari^1,^*, Subhashis Pal^5,^*, Rakesh Kumar Majhi^1,2,^*, Ashutosh Kumar^1,2,^, Rashmita Das^1,2,^, Abhishek Singh^3,4^,. Pradip K. Maji^6^, Naibedya Chattopadhyay^5^, Luna Goswami^3,4^, Chandan Goswami^1,2,#^.**

**1.** School of Biological Sciences, National Institute of Science Education and Research (NISER) -Bhubaneswar, Jatni, Khurda -752050, Odisha, India.

**2.** Homi Bhabha National Institute, Training School Complex, Anushakti Nagar, Mumbai, 400094, India.

**3.** School of Biotechnology, Kalinga Institute of Industrial Technology, Patia, Bhubaneswar 751024, India.

**4.** School of Chemical Technology, Kalinga Institute of Industrial Technology, Patia, Bhubaneswar 751024, India.

**5.** Division of Endocrinology and Center for Research in Anabolic Skeletal Target in Health and Illness (ASTHI), Central Drug Research Institute (CDRI), Council of Scientific and Industrial Research (CSIR), Lucknow, 226031, India; AcSIR, CSIR-Central Drug Research Institute Campus, Lucknow, 226031, India.

**6.** Dept. of Polymer & Process Engineering, Indian Institute of Technology Roorkee, Saharanpur Campus, Paper Mill Road, Saharanpur, Uttar Pradesh- 247001, India

♣ = Equal contribution

* = Equal contribution

# = Correspondence ([chandan@niser.ac.in](mailto:chandan@niser.ac.in))


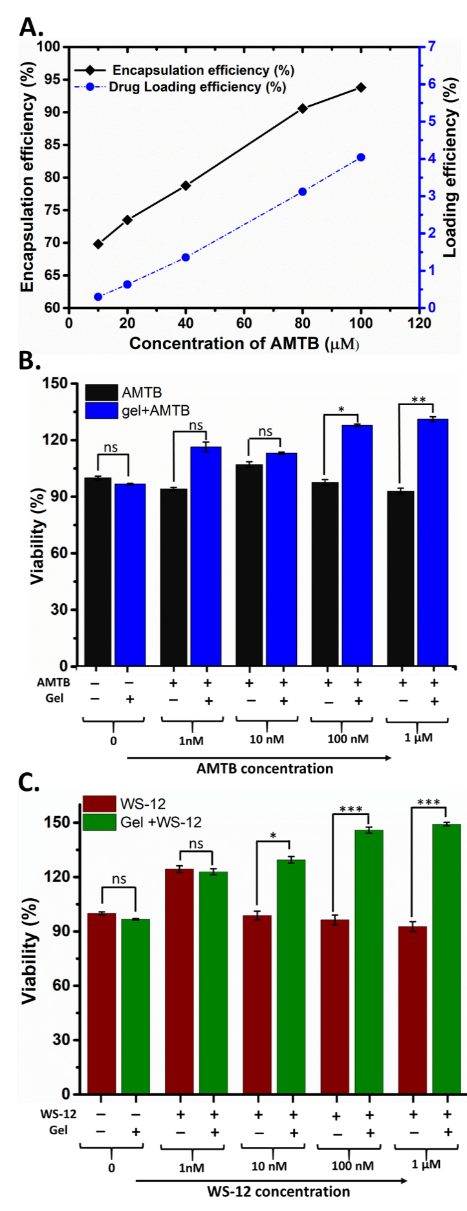


**Supplementary figure 1: Drug release assay and cell proliferation assay.** **A.** Concentration-dependent drug encapsulation efficiency and drug loading efficiency using AMTB is shown. **B-C.** Mouse bone marrow derived mesenchymal stem cells were seeded on only hydrogel or on the drug-coated hydrogels and their survival rate as detected by MTT assay is shown. Cells grown on only glass cover slips is used as a reference.


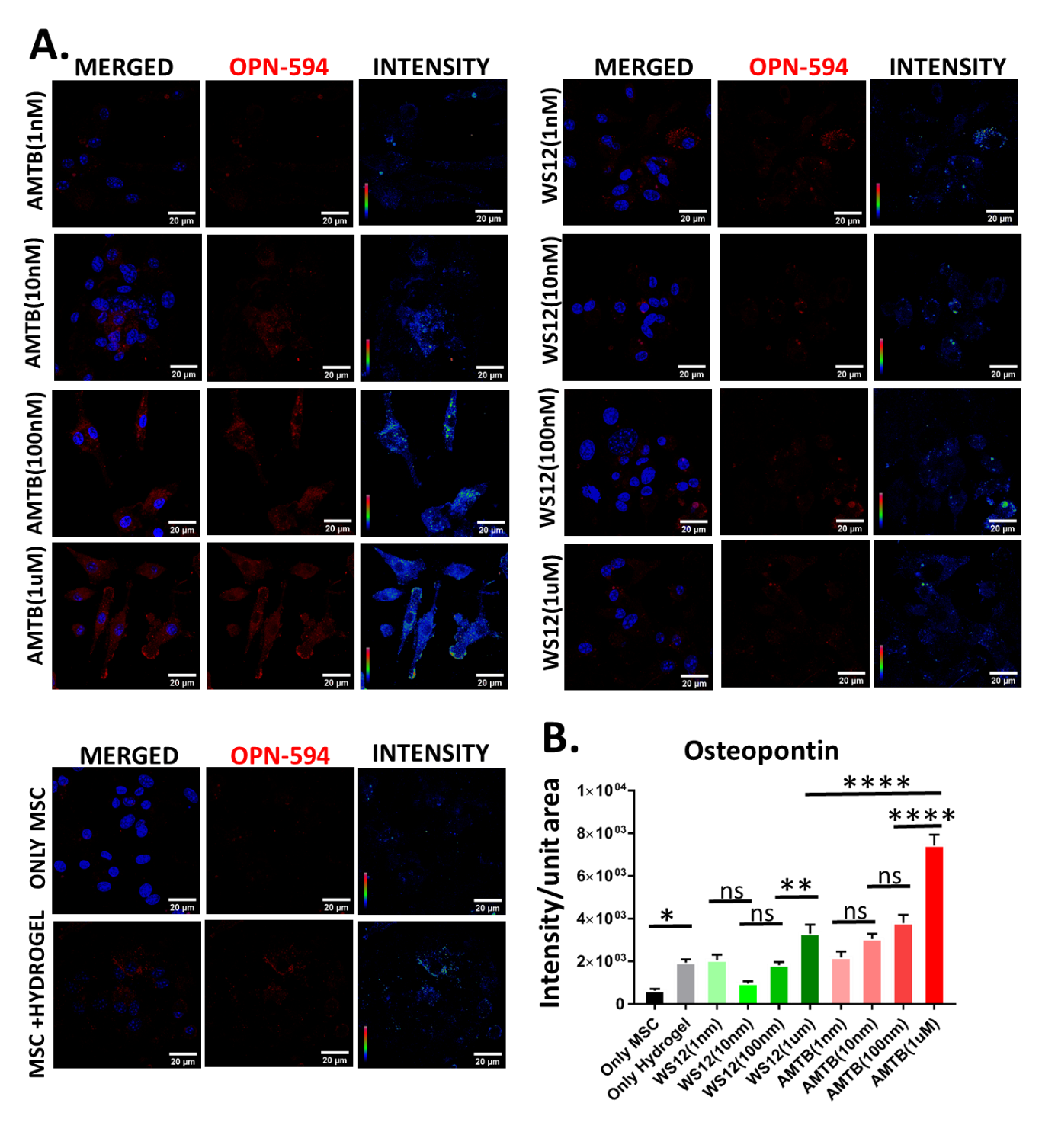


**Supplementary figure 2: Osteopontin expression in MSCs is induced when grown on different surfaces with TRPM8 modulatory drugs**. **A.** Mesenchymal Stem Cells isolated from mouse bone marrow were grown on the hydrogel coated with different concentrations of AMTB (1nm, 10nm, 100nm, 1uM) and WS12 (1nm, 10nm, 100nm, 1uM). After 14 days of growing in mineralization media the cells were fixed and stained with antibody specific for Osteopontin. Dose-dependent increment in Osteopontin expression is observed in case of AMTB-coated hydrogels. Intensity of Osteopontin is represented in pseudo rainbow color. **B**. The intensity of Osteopontin is quantified by using image J software. The expression of OPN is more in AMTB (1uM) in compared to the WS12 and control condition**.**


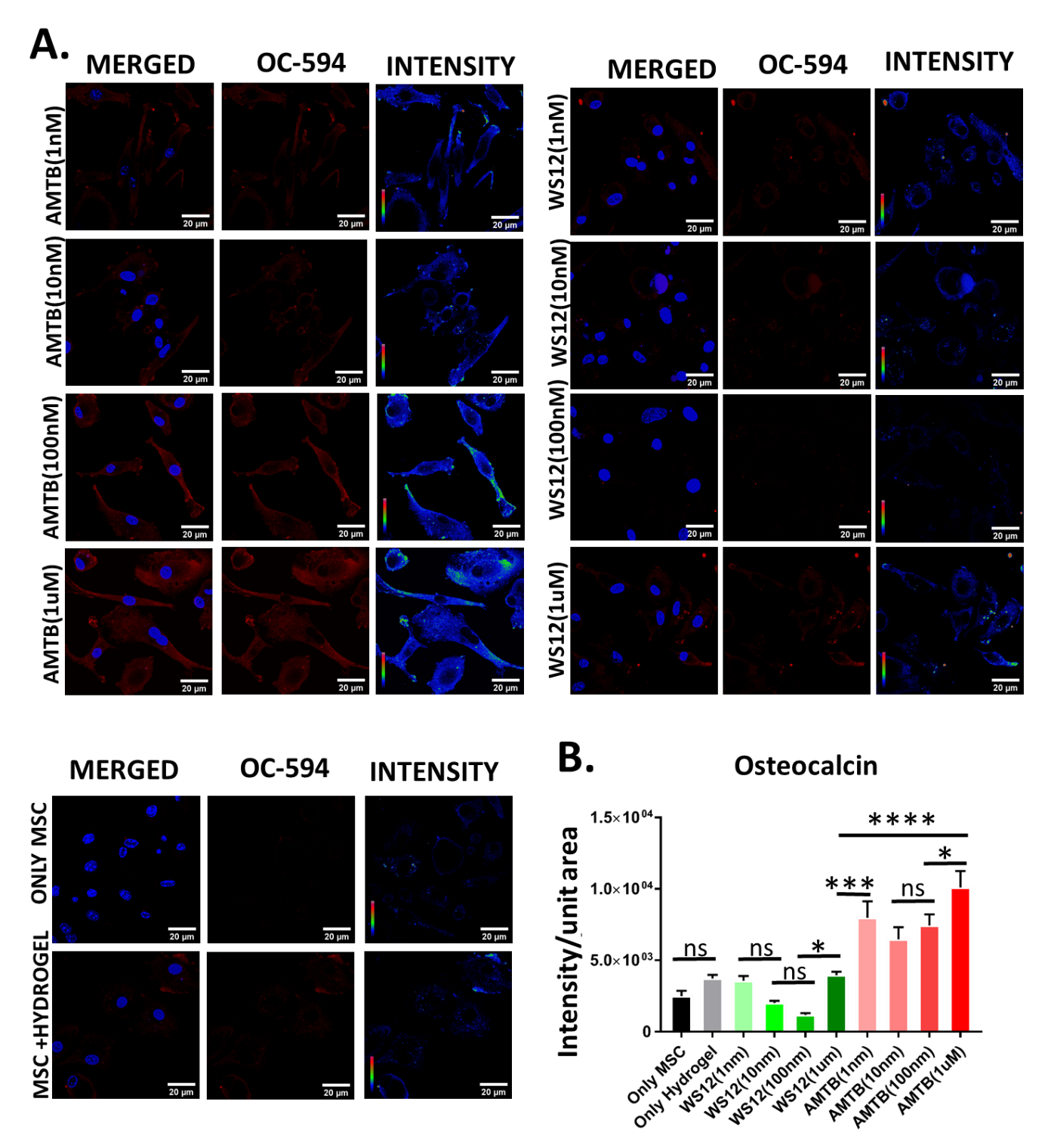


**Supplementary figure 3: Osteocalcin expression in MSCs is induced when grown on different surfaces with TRPM8 modulatory drugs**. **A.** Mesenchymal Stem Cells isolated from mouse bone marrow were grown on the hydrogel coated with different concentrations of AMTB (1nm, 10nm, 100nm, 1uM) and WS12 (1nm, 10nm, 100nm, 1uM). After 14 days of growing in mineralization media the cells were fixed and stained with antibody specific for Osteocalcin. Higher levels of Osteocalcin expression is observed in case of AMTB-coated hydrogels. Intensity of Osteocalcin is represented in pseudo rainbow color. **B**. The intensity of Osteocalcin is quantified by using image J software. The expression of OCN is more in AMTB (1uM) in compared to the WS12 and control condition**.**


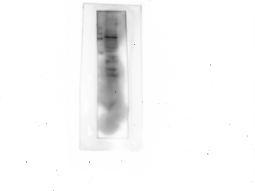


**Supplementary figure 4: Full-size unedited blot of image 1E.**
